# A targeted metabolomics assay for cardiac metabolism and demonstration using a mouse model of dilated cardiomyopathy

**DOI:** 10.1007/s11306-016-0956-2

**Published:** 2016-03-07

**Authors:** James A. West, Abdelaziz Beqqali, Zsuzsanna Ament, Perry Elliott, Yigal M. Pinto, Eloisa Arbustini, Julian L. Griffin

**Affiliations:** The Department of Biochemistry & The Cambridge Systems Biology Centre, University of Cambridge, Tennis Court Road, Cambridge, CB2 1GA UK; The Elsie Widdowson Laboratory, Medical Research Council Human Nutrition Research, 120 Fulbourn Road, Cambridge, CB1 9NL UK; Department of Experimental Cardiology, Academic Medical Centre, Amsterdam, The Netherlands; Heart Hospital, University College London, London, W1G 8PH UK; IRCCS Fondazione Policlinico San Matteo Pavia, Pavia, Italy

**Keywords:** Metabonomics, Tandem mass spectrometry, Lamin A/C, Cardiac disease

## Abstract

Metabolomics can be performed either as an ‘open profiling’ tool where the aim is to measure, usually in a semi-quantitative manner, as many metabolites as possible or perform ‘closed’ or ‘targeted’ analyses where instead a pre-defined set of metabolites are measured. Targeted methods can be designed to be more sensitive and quantitative and so are particularly appropriate to systems biology for quantitative models of systems or when metabolomics is performed in a hypothesis driven manner to test whether a particular pathway is perturbed. We describe a targeted metabolomics assay that quantifies a broad range of over 130 metabolites relevant to cardiac metabolism including the pathways of the citric acid cycle, fatty acid oxidation, glycolysis, the pentose phosphate pathway, amino acid metabolism, the urea cycle, nucleotides and reactive oxygen species using tandem mass spectrometry to produce quantitative, sensitive and robust data. This assay is illustrated by profiling cardiac metabolism in a lamin A/C (Lmna) mouse model of dilated cardiomyopathy (DCM). The model of DCM was characterised by increases in concentrations of proline and methyl-histidine suggestive of increased myofibrillar and collagen degradation, as well as decreases in a number of citric acid cycle intermediates and carnitine derivatives indicating reduced energy metabolism in the dilated heart. These assays could be used for any other cardiac or cardiovascular disease in that they cover central core metabolism and key pathways involved in cardiac metabolism, and may provide a general start for many mammalian systems.

**Electronic supplementary material:**

The online version of this article (doi:10.1007/s11306-016-0956-2) contains supplementary material, which is available to authorized users.

## Introduction

Metabolomics is the global profiling of the metabolic composition of a cell, tissue, organism or biofluid and has wide ranging applications in many fields including biology, medicine, functional genomics, the pharmaceutical industry, and agrochemicals (Nicholson et al. 1999; Fiehn 2002; Goodacre et al. 2004; Mayr 2008; Griffin et al. 2011; Heather et al. 2013). Mass spectrometry based approaches are increasingly being used in metabolomics to provide a snap shot of global metabolism in biological studies, reflecting the sensitivity of the approach to detect a wide range of different chemicals. The analyses can be separated into two differing philosophies. Open profiling based metabolomics are non-targeted approaches which aim to measure as many metabolites as possible using an assay that is a compromise for a wide ranging list of metabolites. While popular in biomarker discovery studies [for example (Hodson et al. 2007; Dunn et al. 2008; Zhang et al. 2012)] they are at best semi-quantitative, often performed with a limited number of standards using mass analysers that are prone to sensitivity drift but good at analysing a wide range of metabolites. Limits of detection are reduced by both the compromised nature of the assay (i.e. optimisation is performed for a diverse range of chemicals) and the dynamic range of the assay, although it should be noted that many high resolution mass spectrometers can routinely achieve a dynamic range of 10^4^. Furthermore, open profiling techniques necessitate the use of multivariate statistics, and for mass spectrometry complex software based alignment tools for analysing chromatographic domains and access to detailed databases to aid structure elucidation if the user is to consider the majority of the metabolites detected (Kind et al. 2009; Wishart 2009; Tautenhahn et al. 2012).

Alternatively, targeted methods have increased in popularity, particularly in systems biology, systems medicine and biomarker validation where quantification is important. These methods target a limited number of metabolites, often relying on triple quadrupole mass spectrometry, and because of this targeting have improved limits of detection and quantification compared with open profiling approaches. Data analysis for these types of datasets is also simplified as the user already knows what metabolites should have been detected and can set up a specific processing method for the assay which identifies metabolites according to fragmentation pattern and retention time. Such approaches have been used to model metabolism in *E. Coli* (Bennett et al. 2009), follow metabolic changes in myocardial infarction and insulin resistance/type 2 diabetes (Lewis et al. 2008; Wang et al. 2011; Wang-Sattler et al. 2012) and perform genome wide association studies (GWAS) (Gieger et al. 2008). To further enhance the robustness and reliability of the method, chromatography can be optimized prior to mass spectrometry analysis to ensure the separation of isobaric species (for example, the metabolites leucine and isoleucine) or species that fragment in such a way that they resemble other species (for example ATP may fragment under certain conditions to resemble ADP or AMP in terms of the ions produced). In this manuscript we detail a targeted analysis of cardiac metabolism, that while appropriate to a range of cardiac diseases we have tested on a mouse model of inherited cardiomyopathies.

Inherited cardiomyopathies are diseases caused by a single mutation of a gene that subsequently affects the structure and function of the heart. Two common forms are hypertrophic cardiomyopathies (HCM) where the heart increases in size as a result of increased muscle wall thickness and dilated cardiomyopathy (DCM) where the increase in heart size is not accompanied by an increase in wall thickness. The incidence rate of DCM is 1 in 2500, and is the commonest cause of cardiac transplantation and death for non-ischaemic heart failure in young adolescents and adults (Taylor et al. 2006). Over 50 genes are involved in HCM and DCM, producing heterogeneous phenotypes for the diseases (Judge 2009). Furthermore, the observed phenotypes are also complicated by the fact that these mutations interact with the wider genome of the individual, further increasing the heterogeneity of the disease. Currently, less than 1 % of those with familial DCM are genotyped in part because of the large number of genes and mutations involved. Thus, there is a clinical need for biomarkers that can identify individuals with DCM and HCM. In addition such biomarkers could be used to follow treatment efficacy.

When designing a targeted metabolomics assay for an inherited cardiomyopathy it should be noted that a number of metabolic abnormalities have been previously associated with both DCM and HCM. DCM has been associated with the generation of reactive oxygen species (ROS), particularly as a result of mitochondrial stress (Charniot et al. 2011; Kitajima et al. 2011; Lu et al. 2012). In the heart one of the major anti-oxidants is glutathione, while ROS will oxidize certain nucleotides and amino acids which can act as surrogate markers of ROS damage (Stadtman and Levine 2003). In addition alterations in substrate selection (Taha and Lopaschuk 2007), and in particular altered fatty acid β-oxidation have been reported in both DCM (Feinendegen et al. 1995) and HCM (Nakamura et al. 2000), while mutations associated with 5′ adenosine monophosphate-activated protein kinase (AMPK), a master regulator of metabolism, have been linked to a range of cardiomyopathies including diabetic cardiomyopathy, DCM and HCM (Dolinsky and Dyck 2006; Taha and Lopaschuk 2007). The heart has a high rate of β-oxidation, and the carnitine shuttle transports fatty acids into the mitochondria across the inner mitochondrial membrane for oxidation as acyl-carnitines. Thus, the measurement of tissue acyl-carnitines can determine mitochondrial function and substrate selection. Furthermore, phosphorylated nucleotides represent both the energy status of the heart (ATP, ADP and AMP), and important regulatory molecules used to determine substrate selection (cAMP).

Both HCM and DCM ultimately progress to the failing heart, and in this state it has been observed that there is a switch from the adult isoforms of key metabolic enzymes to the fetal isoforms (Razeghi et al. 2002). These enzymes include glucose transporters and mitochondrial carnitine palmitoyl transferase-1, with these changes responsible for a decrease in fatty acid oxidation and an increase in glycolysis in the failing heart.

Here we describe a series of targeted assays for the metabolic profiling of cardiac tissue. These assays are robust in performance, and sensitive in terms of limit of detection and quantitative. The assays target key metabolites involved in the core pathways involved in energy production in the heart (citric acid cycle, glycolysis and β-oxidation), ROS to monitor oxidative damage in the cell and protein turnover (amino acids), providing analysis of over 130 metabolites in total. We illustrate their use by examining the metabolic alterations associated with Lmna knockout mouse (Sullivan et al. 1999) compared with wildtype controls. Although developed to profile tissue from DCM and HCM patients and the associated animal models, these assays could be used for other cardiac disorders, and may provide a useful starting point for targeted mammalian metabolomics.

## Materials and methods

### Chemicals reagents and chromatography columns

LC–MS grade solvents and mobile phase additives were obtained from Sigma Aldrich (Gillingham, Dorset, UK). All standards for optimisation and quantitation including the [U–^13^C] succinate used as an internal standard were also obtained from Sigma Aldrich with the exception of the [U–^13^C, U–^15^N] glutamate and the mixed standard of eight deuterated acyl carnitines that was obtained from Cambridge Isotope Laboratories (Andover, MA, USA). The ZIC-HILIC sulfo betaine column was obtained from VWR (Radnor, PA, USA), the Synergi Polar RP column from Phenomenex (Torrance, CA, USA) and the HSS T3 column from Waters (Milford, MA, USA).

### Animals

Lmna knockout mice on a C57BL6/J background were obtained from a stable colony at the Academic Medical Center in Amsterdam which was generated from a previously described mouse model (Sullivan et al. 1999). For genotyping, genomic DNA was isolated from mice toe biopsies and analysed by PCR. Mutant mice and wild type littermates (C57BL6/J) were studied according to protocols approved by the institutional Animal Ethics Committee at Acadamic Medical Center Amsterdam. All animal experiments were performed in accordance with Dutch law on care and use of experimental animals. Lmna homozygous, heterozygous and wildtype male mice were killed at the ages of 2, 5 and 40 weeks (n = 8 per experimental group). Hearts were quickly harvested, rinsed in PBS, snap frozen in liquid nitrogen and stored at −80 °C until extraction of metabolites.

### Extraction of metabolites from heart tissue

Metabolites were extracted using the methanol/chloroform method described by Le Belle et al. (2002). In brief 50 mg of frozen heart tissue was ground with dry ice in a pestle and mortar and placed inside 2 ml flat-bottomed screw cap tubes. 600 µl of ice cold 2:1 methanol:chloroform was added. After the addition of stainless steel balls, the samples were put into a tissue lyser (Qiagen, Hilden, Germany) for 10 min at 25 Hz to ensure optimum extraction. 200 µl of water and 200 µl of chloroform were added and the samples thoroughly vortexed before centrifugation at 13,200 rpm for 25 min. After centrifugation the aqueous (top layer) and organic (bottom layer) fractions were separated and aliquoted into separate tubes. A further 600 µl of 2:1 methanol: chloroform was added to the original tube and the extraction repeated as above. The aqueous and lipid extracts were dried under nitrogen at room temperature for about 3 h in a fume hood. Both were stored at −20 °C prior to analysis.

### Instrumentation and mass spectrometry parameter optimisation

All analyses were carried out using a Quattro Premier XE quadrupole mass spectrometer coupled to an Acquity ultra performance liquid chromatography (UPLC) system from Waters Ltd. (Atlas Park, Manchester, UK). Compounds were optimised for tandem MS analysis by preparing individual standard solutions at 1 µM in the running buffer for the relevant chromatographic assay, and directly infused for parameter optimisation. Optimum mass spectrometry parameters and mass transitions were obtained by using the automatic optimisation protocols of MassLynx™ (Version 1.4, Waters) and for situations where no standards were available, mass transitions and mass spectrometry parameters were inferred from the parameters of known analogues.

### Analysis of HILIC mode polar compounds measured in positive ion mode including nucleotides and acyl CoAs

One half of the aqueous extract was dissolved in 150 µl of 70:30 acetonitrile:water containing 20 µM deoxy-glucose 6 phosphate and 20 µM [U–^13^C, ^15^N] glutamate. The resulting solution was vortexed, then sonicated for 15 min followed by centrifugation at 15,000 rpm with a bench top centrifuge to pellet any remaining undissolved material. The supernatant was transferred into a 300 µl vial (Agilent, Santa Clara, CA, USA) and capped ready for analysis. For chromatography on the UPLC system, the strong mobile phase (A) was 100 mM ammonium acetate, the weak mobile phase was acetonitrile (B) and the LC column used was the ZIC-HILIC column from Sequant (100 mm × 2.1 mm, 5 µm). The following linear gradient was used: 5 % A in acetonitrile was increased to 50 % A over 12 min with re-equilibration for a further 3 min. The total run time was 15 min, the flow rate was 0.3 ml/min and the injection volume was 2 µl. The metabolites were separated into two functions consisting of 32 and 11 MRMs owing to software limitations governing the number of MRMs allowed per function. Mass spectrometry parameters were the following: positive ion mode, a desolvation temperature of 300 °C, a source temperature of 110 °C, an ion spray voltage of 3.5 kV and a dwell time of 10 ms for each analyte. Compound specific parameters such as cone voltage and collision energy are listed in Table 1.

**Table 1:** Compound specific mass spectrometry parameters

Compound	Ion mode	Parent mass (*m/z*)	Daughter mass (*m/z*)	Declustering potential (V)	Collision energy (eV)	Column used	RT (min)
^13^C_5_^15^N_1_ glutamate (IS)	+	154.1	89.0	46	21	ZIC HILIC	5.72
^13^C_5_^15^N_1_ glutamate dibutyl ester (IS)	+	266.2	163.1	25	15	HSS T3	5.86
2-Phosphoglycerate	−	184.9	78.8	−35	−20	BEH amide	2.41
3-Phosphoglycerate	+	187.0	105.0	46	11	BEH amide	2.44
Acetyl CoA	+	810.0	303.2	81	39	ZIC-HILIC	5.75
Aconitate	−	173.0	85.0	−35	−17	BEH amide	1.89
Adenine	+	136.0	119.0	126	29	ZIC-HILIC	0.83
Adenosine	+	268.1	136.1	51	23	ZIC-HILIC	1.12
Adenosyl methionine	+	399.0	250.1	86	21	ZIC-HILIC	7.39
ADP	+	428.0	136.0	86	27	ZIC-HILIC	6.58
Ala butyl ester	+	146.1	44.1	25	15	HSS T3	2.23
AMP	+	348.1	136.0	51	23	ZIC-HILIC	6.03
Anserine butyl ester	+	297.2	226.2	30	20	HSS T3	1.43
Arg butyl ester	+	231.2	70.1	25	15	HSS T3	1.11
Asn butyl ester	+	188.9	73.8	20	20	HSS T3	1.58
Asp dibutyl ester	+	246.2	144.1	25	15	HSS T3	5.61
ATP	+	508.0	136.0	150	28	ZIC-HILIC	6.92
Betaine butyl ester	+	173.9	117.9	25	20	HSS T3	2.40
C10 carnitine butyl ester	+	372.3	85.0	35	25	Phenyl ether	2.30
C10:1 carnitine butyl ester	+	370.3	85.0	35	25	Phenyl ether	2.12
C10:2 carnitine butyl ester	+	368.3	85.0	35	25	Phenyl ether	2.00
C12 carnitine butyl ester	+	400.3	85.0	35	25	Phenyl ether	2.81
C12:1 carnitine butyl ester	+	398.3	85.0	35	25	Phenyl ether	2.59
C14 carnitine butyl ester	+	428.4	85.0	35	25	Phenyl ether	3.98
C14:1 carnitine butyl ester	+	426.4	85.0	35	25	Phenyl ether	3.51
C14:2 carnitine butyl ester	+	424.3	85.0	35	25	Phenyl ether	3.05
C14-OH carnitine butyl ester	+	444.4	85.0	35	25	Phenyl ether	3.30
C16 carnitine butyl ester	+	456.4	85.0	35	25	Phenyl ether	4.47
C16:1 carnitine butyl ester	+	454.4	85.0	35	25	Phenyl ether	4.25
C16:1-OH carnitine butyl ester	+	470.4	85.0	35	25	Phenyl ether	3.60
C16:2 carnitine butyl ester	+	452.4	85.0	35	25	Phenyl ether	3.90
C16-OH carnitine butyl ester	+	472.4	85.0	35	25	Phenyl ether	4.01
C18 carnitine butyl ester	+	484.4	85.0	35	25	Phenyl ether	4.83
C18:1 carnitine butyl ester	+	482.4	85.0	35	25	Phenyl ether	4.65
C18:1-OH carnitine butyl ester	+	498.4	85.0	35	25	Phenyl ether	3.85
C18:2 carnitine butyl ester	+	480.4	85.0	35	25	Phenyl ether	4.34
C18:2-OH carnitine butyl ester	+	496.4	85.0	35	25	Phenyl ether	3.55
C18-OH carnitine butyl ester	+	500.4	85.0	35	25	Phenyl ether	4.15
C2 carnitine butyl ester	+	260.2	85.0	35	25	Phenyl ether	0.60
C20 carnitine butyl ester	+	512.4	85.0	35	25	Phenyl ether	5.11
C20:1 carnitine butyl ester	+	510.4	85.0	35	25	Phenyl ether	4.92
C20:2 carnitine butyl ester	+	508.4	85.0	35	25	Phenyl ether	4.70
C3 carnitine butyl ester	+	274.2	85.0	35	25	Phenyl ether	0.75
C4 carnitine butyl ester	+	288.2	85.0	35	25	Phenyl ether	0.92
C4 dicarboxyl carnitine dibutyl ester	+	374.3	85.0	35	25	Phenyl ether	1.43
C5 carnitine butyl ester	+	302.3	85.0	35	25	Phenyl ether	1.15
C5 dicarboxyl carnitine dibutyl ester	+	388.3	85.0	35	25	Phenyl ether	1.67
C5:1 carnitine butyl ester	+	300.2	85.0	35	25	Phenyl ether	1.00
C5-OH carnitine butyl ester	+	318.2	85.0	35	25	Phenyl ether	0.64
C6 carnitine butyl ester	+	316.3	85.0	35	25	Phenyl ether	1.42
C6 dicarboxyl carnitine dibutyl ester	+	402.3	85.0	35	25	Phenyl ether	2.82
C8 carnitine butyl ester	+	344.3	85.0	35	25	Phenyl ether	1.89
C8 dicarboxyl carnitine dibutyl ester	+	430.4	85.0	35	25	Phenyl ether	4.02
C8:1 carnitine butyl ester	+	342.3	85.0	35	25	Phenyl ether	1.87
C8-OH carnitine butyl ester	+	361.3	85.0	35	25	Phenyl ether	1.20
cAMP	+	330.1	136.1	71	31	ZIC-HILIC	1.85
Carnosine butyl ester	+	283.2	109.9	25	30	HSS T3	1.32
CDP	−	402.0	78.9	−25	−80	ZIC-HILIC	7.14
CDP-choline	+	489.1	184.1	76	47	ZIC-HILIC	6.85
cGMP	+	346.1	152.1	41	23	ZIC-HILIC	3.22
Citrate tributyl ester	+	361.2	185	22	15	HSS T3	9.39
Citrulline butyl ester	+	232.1	69.9	20	25	HSS T3	2.00
CMP	+	324.1	112.0	71	17	ZIC-HILIC	6.34
CTP	−	481.9	158.8	−85	−34	ZIC-HILIC	7.39
Cystine dibutyl ester	+	353.2	73.9	30	35	HSS T3	3.60
Cytidine	+	244.1	112.0	61	15	ZIC-HILIC	1.73
Cytosine	+	112.0	95.0	136	25	ZIC-HILIC	1.39
d3 C16 carnitine butyl ester	+	459.4	85.0	35	25	Phenyl ether	4.47
d3 C2 carnitine butyl ester	+	263.2	85.0	35	25	Phenyl ether	0.60
d3 C3 carnitine butyl ester	+	277.2	85.0	35	25	Phenyl ether	0.75
d3 C4 carnitine butyl ester	+	291.2	85.0	35	25	Phenyl ether	0.92
d3 C8 carnitine butyl ester	+	347.3	85.0	35	25	Phenyl ether	1.89
d9 C14 carnitine butyl ester	+	437.4	85.0	35	25	Phenyl ether	3.98
d9 C5 carnitine butyl ester	+	311.3	85.0	35	25	Phenyl ether	1.15
d9 carnitine butyl ester	+	227.2	85.0	35	25	Phenyl ether	0.40
Deoxy glucose 6 phosphate (IS)	−	243.0	96.9	−62	−20	BEH Amide	1.95
Dihydroxyacetonephosphate	−	168.9	96.9	−65	−12	BEH amide	2.45
FAD	+	786.1	348.0	191	29	BEH amide	1.23
Free carnitine butyl ester	+	218.2	85.0	35	25	Phenyl ether	0.40
Fructose bisphosphate	−	339.0	96.9	−30	−24	BEH amide	3.45
Fumarate butyl ester	+	173.2	173.2	30	5	HSS T3	4.00
GDP	+	444.0	152.0	91	23	ZIC-HILIC	6.99
Gln butyl ester	+	203.0	83.8	20	20	HSS T3	1.72
Glu dibutyl ester	+	260.2	158.1	25	15	HSS T3	5.86
Glucose 6 phosphate/Fructose 6 phosphate	−	259.0	96.9	−60	−18	BEH amide	2.35/2.79
Gly butyl ester	+	132.1	76.0	25	15	HSS T3	1.67
GMP	+	364.2	152.1	61	19	ZIC-HILIC	6.68
GSH	+	308.1	179.0	46	17	ZIC-HILIC	7.49
GSSG	+	613.1	355.0	126	31	ZIC-HILIC	9.05
GTP	+	523.9	152.0	151	27	ZIC-HILIC	7.26
Guanine	+	152.0	134.9	66	25	ZIC-HILIC	1.46
Guanosine	+	284.1	152.1	16	17	ZIC-HILIC	1.79
His butyl ester	+	212.1	110.1	25	15	HSS T3	0.82
Leu/Ileu butyl ester	+	188.2	86.1	25	15	HSS T3	4.72/4.64
Lys butyl ester	+	203.2	84.1	25	15	HSS T3	0.92
Malonyl CoA	+	854.0	347.1	81	41	ZIC-HILIC	6.79
Met butyl ester	+	206.1	104.1	25	15	HSS T3	4.04
Methyl Cytosine	+	136.0	109.1	116	25	ZIC-HILIC	1.21
Methyl Histidine butyl ester	+	226.0	95.8	35	25	HSS T3	0.84
NAD	+	664.0	427.9	111	35	ZIC-HILIC	5.89
NADP	−	741.9	619.8	−65	−22	ZIC-HILIC	6.92
*o*-Hydroxy Tyr butyl ester	+	254.1	152.0	26	17	HSS T3	4.20
*o*-Nitro tyrosine butyl ester	+	283.2	181.0	26	17	HSS T3	4.35
Orn butyl ester	+	189.0	69.9	20	20	HSS T3	0.72
Oxaloacetate	−	131.0	87.0	−65	−10	BEH amide	0.70
Oxo-methionine	+	165.0	105.0	51	7	ZIC-HILIC	4.75
PCr	−	210.0	78.9	−55	−18	BEH amide	1.89
PEP	−	166.9	78.9	−40	−16	BEH amide	1.95
Phe butyl ester	+	222.2	120.1	25	15	HSS T3	5.17
Pro butyl ester	+	172.1	70.1	25	15	HSS T3	2.74
Pyruvate	−	87.0	43.0	−45	−10	BEH amide	0.60
*S*-adenosyl-*L*-homocysteine	+	385.1	136.1	91	23	ZIC-HILIC	3.91
Ser butyl ester	+	162.1	60.0	25	15	HSS T3	1.69
Succinate	−	117.0	73.0	−35	−16	BEH Amide	1.20
Thr butyl ester	+	176.1	74.1	25	15	HSS T3	2.11
Trp butyl ester	+	261.2	159.1	20	20	HSS T3	5.68
Tyr butyl ester	+	238.1	136.1	25	15	HSS T3	4.01
UDP	−	402.9	78.9	−45	−86	ZIC-HILIC	6.39
UMP	+	325.1	96.9	106	17	ZIC-HILIC	6.13
Uracil	+	112.9	70.1	111	23	ZIC-HILIC	0.91
Uridine	+	245.1	112.9	81	17	ZIC-HILIC	1.17
UTP	−	482.9	158.9	−45	−34	ZIC-HILIC	6.93
Val butyl ester	+	174.2	72.1	25	15	HSS T3	3.80
α ketoglutarate	−	145.0	101.0	−40	−12	BEH Amide	1.35

The table shows ionisation mode, mass transitions (parent and daughter masses) and retention times as well as declustering potentials and the collision energies required for each analyte

### Analysis of HILIC mode polar compounds measured in negative ion mode including glycolytic intermediates and TCA cycle intermediates

The sample from the positive ion mode analysis was recovered and analysed in a second UPLC chromatography assay. The strong mobile phase (A) was 10 mM ammonium acetate with 0.05 % ammonium hydroxide and the weak mobile phase was acetonitrile (B), and the LC column used was a BEH amide HILIC column (100 × 2.1 mm, 1.7 µm; Waters Ltd). The following linear gradient was used: 30 % A was held for 2 min followed by a linear gradient to 50 % A at 7 min with further re-equilibration for 3 min, the total run time was 10 min. Mass spectrometry parameters were the following: negative ion mode, a desolvation temperature of 300 °C, a source temperature of 110 °C, an ion spray voltage of 3.0 kV and a dwell time of 10 ms for each analyte. Compound specific parameters such as cone voltage and collision energy were optimised according to the protocol above and are listed in Table 1. Several compounds ionised in both positive and negative mode and so were included in both types of HILIC analyses.

### Analysis of amino acids

The remaining sample from the HILIC analysis was thoroughly dried under nitrogen and derivatised with 200 µl of 3 M HCl in BuOH for 15 min at 65 °C. After further drying, the sample was reconstituted in 9:1 0.1 % formic acid in water/acetonitrile and sonicated to ensure solvation of the amino acid derivatives. Samples were analysed on the UPLC interfaced with the triple quadrupole LC–MS/MS. The strong mobile phase used for analysis was acetonitrile (B) and the weak mobile phase was 0.1 % formic in water (A). The analytical UPLC gradient used a HSS T3 column (100 mm × 2.1 mm, 1.7 µm) from Waters Ltd with 5 % B in 0.1 % formic acid at 0 min followed by a linear gradient to 40 % B after 7 min and another gradient to 100 % B at 10 min followed by re-equilibration for 3 min. The total run time was 13 min and the flow rate was 0.5 ml/min with an injection volume of 2 µl. The mass spectrometry parameters were: source temperature 150 °C, desolvation temperature 350 °C, capillary voltage 3.5 kV and 700 l/h of desolvation gas, all other parameters were compound specific and are detailed in Table 1.

### Analysis of acyl carnitines

200 µl of a mixed standard of eight deuterated carnitines (Cambridge Isotope Laboratories, Andover, MA, USA) was diluted into 25 ml of acetonitrile. 200 µl of this solution was added to one half of the organic fraction from the original tissue extraction and this was dried down under nitrogen and derivatised with 3 M HCl in butanol for 15 min at 65 °C. The resulting mixture was dried under nitrogen again and mixed with one half of the sample remaining from the amino acid analysis. This mixture was dried a further time and finally reconstituted in 4:1 acetonitrile/0.1 % formic acid in water followed by sonication to dissolve all species present. Samples were analysed by LC–MS/MS. The strong mobile phase used for analysis was acetonitrile with 0.1 % formic acid (B) and the weak mobile phase was 0.1 % formic acid in water (A). The analytical UPLC gradient used a Synergi Polar RP phenyl ether column (100 mm × 2.1 mm, 2.5 µm) from Phenomenex with 30 % B in 0.1 % formic at 0 min followed by a linear gradient to 100 % B for 3 min and held at 100 % B for the next 5 min with a further 2 min re-equilibration. The total run time was 10 min and the flow rate was 0.5 ml/min with an injection volume of 2 µl. The mass spectrometry parameters were: source temperature 150 °C, desolvation temperature 350 °C, capillary voltage 3.5 kV and 500 l/h of desolvation gas, all other parameters were compound specific and are detailed in Table 1.

### Data analysis

Data were processed using QuanLynx within MassLynx (version 1.4; Waters Corp., Milford, USA). The data were imported into SIMCA-P+ version 12.0 (Umetrics, Umeå, Sweden) for multivariate analysis by principal components analysis (PCA), partial least squares (PLS) and partial least squares discriminate analysis (PLS-DA). Data sets were analysed using PCA for a global visualisation of the dominant trends in the datasets followed by PLS and PLS-DA to examine specified clustering or trends. In an ideal world the supervised approaches would be cross validated using a train and test routine where 2/3 of the data are used to train the models produced and the further 1/3 to test the model robustness. However, this is neither cost effective nor ethical for many animal studies. Instead, to limit animal numbers used in the study we used a random permutation test. In this process the percentage variance explained (R^2^) and goodness of fit (Q^2^) of a model is compared with models generated where the class membership has been randomly permuted. If the true model is significantly better than the random models one has confidence in the overall robustness of the original model. Student’s t tests and other univariate approaches were carried out using Excel™ (Microsoft Corp.), with a significance set to p < 0.05.

## Results and discussion

**Fig. 1:**
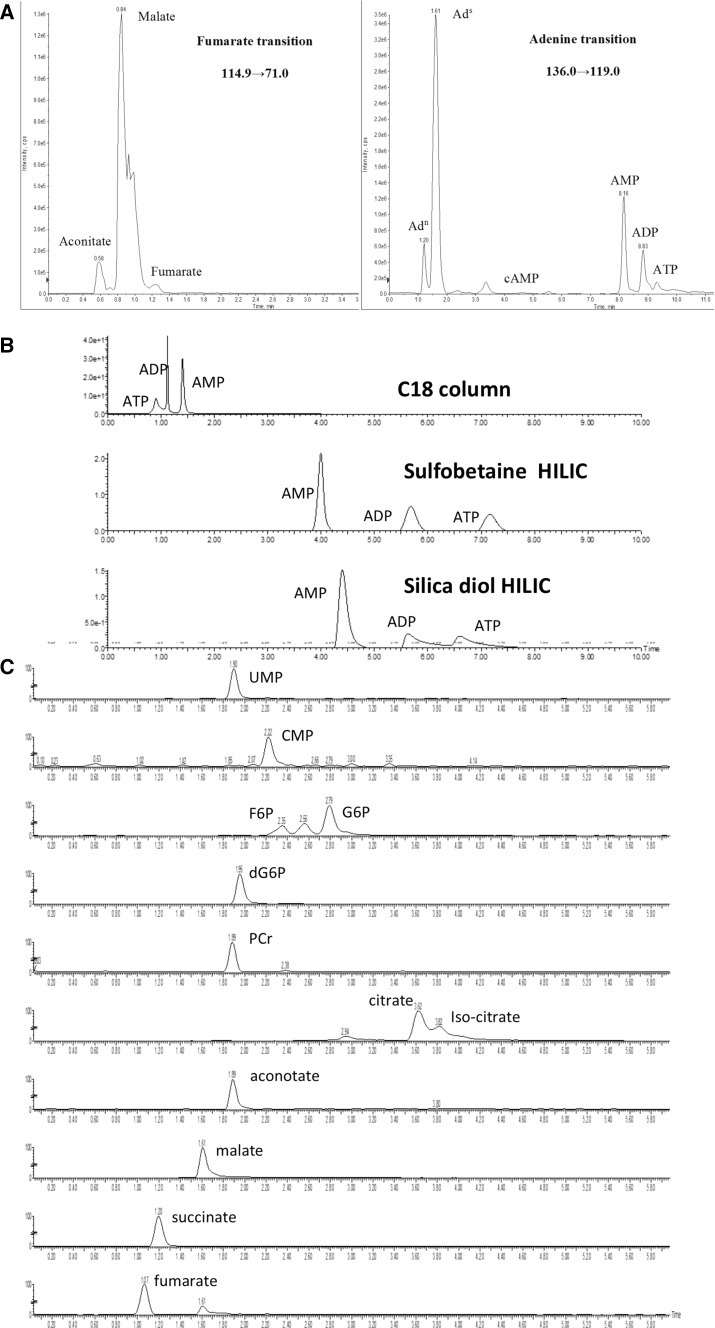
Optimisation of the LC–MS/MS method for studying cardiac metabolism. **a** Fumarate and adenine transitions using polar reverse phase chromatography for fumarate (Polar RP (5 × 2.1 mm, 2.5 µm), Phenomenex) and HILIC chromatography for adenine from aqueous heart tissue extracts. **b** Three UV chromatograms of a mixed standard of AMP, ADP and ATP at 10 µM showing different approaches to the separation of highly polar analytes. The C18 method used a C18 column (100 × 2.1, 1.7 µm; HSS T3 column, Waters) using an isocratic 5 min gradient of 5 % acetonitrile in 0.1 % formic acid at a flow rate of 400 µl/min. The sulfobetaine HILIC method used a ZIC-HILIC column (100 × 2.1, 3.5 µm; Merck) and an isocratic gradient of 30 % 100 mM NH_4_OAc in acetonitrile at a flow rate of 200 µl/min. The silica diol HILIC method used a HILIC column (100 × 2.1, 2.5 µm, Phenomenex) and an isocratic gradient of 30 % 20 mM NH_4_OAc in acetonitrile at a flow rate of 300 µl/min. All analyses were measured at λ = 260 nm. **c** A series of extracted ion chromatograms showing negative ion mode compounds in an aqueous extract of a mouse heart tissue sample separated on a BEH amide column. (100 × 2.1 mm, 1.7 µm; Waters Ltd.)

To optimise a method for the analysis of a large range of metabolites a suitable column must be identified to provide good chromatographic separation, minimize suppression effects (the ability of one metabolite to reduce the signal from another metabolite in the mass spectrometer) and aid the detection of metabolites that might undergo source fragmentation. If a given compound fragments into its metabolic precursor in the source of the mass spectrometer then for a given mass transition several compounds might be detected. Phosphate species and citric acid cycle intermediates are a particular problem as phosphate and water, respectively, can be lost when compounds undergo electrospray ionisation (ESI). Figure 1a shows how source fragmentation can cause a single mass channel to contain all analytes that break down into the compound investigated. All adenine-containing nucleotides lose their phosphate or sugar groups and aconitate and malate lose carbon dioxide and water, respectively, to yield fumarate during ionisation. It was therefore necessary to use a chromatography approach that separated all of these compounds. The nucleoside phosphates proved particularly difficult as they tend to be poorly retained on reverse phases and too well retained on HILIC phases giving rise to poor peak shape and reproducibility. Figure 1b shows a comparison of C18, HILIC silica diol and HILIC zwitterionic (sulfobetaine) phases for the separation of AMP, ADP and ATP. The symmetry of the peak shape and the efficient separation in the ZIC-HILIC analysis showed that this column was ideal for the analysis of highly polar compounds.

Several of the highly polar analytes such as citric acid cycle intermediates will only ionise in negative ion mode in the source of the mass spectrometer. Negative ion mode often requires the use of alkaline mobile phase additives such as ammonium hydroxide but ZIC-HILIC cannot be run at alkaline pH. Thus, a BEH amide HILIC column was used. The bridged ethyl linkage is stable over a pH range of 2–12 and so is ideal for use with alkaline pH buffers. Figure 1c shows a range of negative ion mode aqueous metabolites in an aqueous mouse heart tissue extract measured on a BEH amide HILIC column. The HILIC chromatography could separate similar polar compounds, including isomers such as glucose 6-phosphate and fructose 6-phosphate where the transition 259 > 97 shows three peaks eluting in order of polarity with the first and last peaks being fructose 6 phosphate and glucose 6 phosphate, respectively, with the middle peak most likely being glucose 1 phosphate (not analysed).

**Fig. 2:**
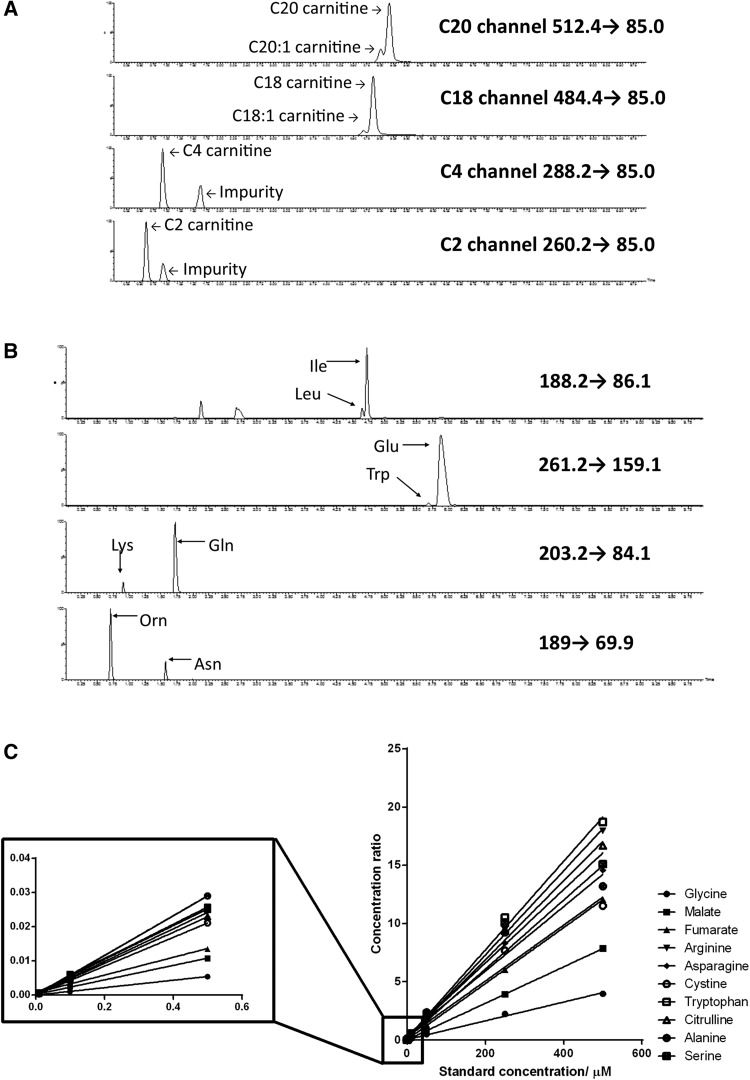
LC–MS/MS analysis of butylated acyl carnitines and amino acids. **a** Four extracted ion chromatograms of a heart tissue extract measured using a Phenomenex Synergi Polar RP column. This figure demonstrates the need for specificity when conducting acyl carnitine analysis with significant impurities detected in two of the channels. **b** Four extracted ion chromatograms of a heart tissue extract measured using a Waters HSS T3 column showing the requirement for chromatographic separation in order to separate isobaric or near isobaric compounds. **c** A linearity graph showing the response of nine amino acids and fumarate over the range 10 nM to 500 μM. [U–^13^C, ^15^N] glutamate was used as an internal standard

Acylcarnitines play a central role in regulating fatty acid oxidation and mitochondrial metabolism. MS analysis of acylcarnitine derivatives by butanolic HCl is an established protocol which benefits from the improved ionisation and characteristic fragmentation pattern associated with the derivatisation. Typically, acylcarnitine derivatives are measured via direct infusion without any chromatography, but this approach presents problems with robustness and specificity. Mass transitions are not completely specific to a given compound and chromatographic separation is required in order to be sure that a mass transition is measuring the correct compound. Furthermore, with analysing heart tissue, there is the potential for ion suppression from phospholipids associated with cell membranes. Thus, we developed a chromatographic method to separate the acylcarnitines following derivatisation (Fig. 2a).

To test linearity ten matrix-matched internal standard replicates (i.e. labelled standards in a heart tissue extract) were injected alongside 10 standard solutions and found to have average coefficients of variation of 12.3 and 6.3 %, respectively. Linearity was investigated in non matrix-matched solutions using free carnitine, acetyl carnitine and palmitoyl carnitine standards with the appropriate deuterated analogue as internal standard for each compound. Free carnitine and acetyl carnitine were found to be linear in the range 5 nM to 20 μM (R^2^ = 0.971, 0.97, respectively), whereas palmitoyl carnitine was found to be linear in the range 50 nM to 20 μM (R^2^ = 0.98).

As the butylation derivatisation protocol forms an ester with the carboxylate moiety it can be used to form derivatives of any carboxylic acid if they are sufficiently stable to survive the derivatisation process including amino acids and stable oxoacids (e.g. citric acid cycle intermediates). This derivatisation process also aids chromatographic separation and the detection of low concentration metabolites. The length of derivatisation using the butanolic HCl protocol is important, as species such as glutamic acid and citric acid can be derivatised several times due to the presence of more than one carboxyl group and species such as glutamine and asparagine contain acid labile amide moieties that can be transformed to butyl esters on reaction with butanolic HCl. Mixtures of standards were dried and treated with 200 µl of butanolic HCl for 15, 30, 45 and 60 min. The time of 15 min was found to be the best as significant amounts of citrate were derivatised 3 times whereas less than 10 % of asparagine and glutamine was broken down into aspartic acid and glutamic acid butyl esters (data not shown).

The derivatisation method for amino acids and acyl carnitines used in the present study has a venerable history, having been developed for the rapid screening of inborn errors of metabolism nearly 20 years ago (Chance et al. 1996). While it requires extra sample preparation it is a very robust method, showing almost no retention time drift over analytical runs of hundreds of samples and requiring a generic processing method to process samples across numerous analytical runs. Furthermore, the added sensitivity afforded by the derivatisation was necessary on the triple quadrapole we used in the current manuscript for lower concentration metabolites, particularly the species produced from reactions with ROS (e.g. oxo-methionine, o-hydroxy-tyrosine and o-nitro-tyrosine).

Having established a suitable gradient for the analysis of 29 amino acids and two oxo-acids (with particular care being taken to ensure that isomeric compounds such as leucine and isoleucine were separated although it was not possible to separate the methyl histidine amino acids) experiments were carried out to determine the robustness of the assay in a biological matrix and linearity of the compounds investigated. Ten replicates of [U–^13^C, ^15^N] glutamic acid and [U–^13^C, ^15^N] proline spiked samples were extracted with human heart tissue and compared to non-matrix-matched standards. The coefficients of variation for the matrix matched injections were 7.1 and 9.5 % for glutamate and proline, respectively, whereas the non-matrix matched injections showed CVs of 9.0 and 8.5 % for labelled glutamate and proline. All compounds behaved linearly across a physiologically relevant range of concentrations (Fig. 2c; Supplementary Fig. 1a).

**Fig. 3:**
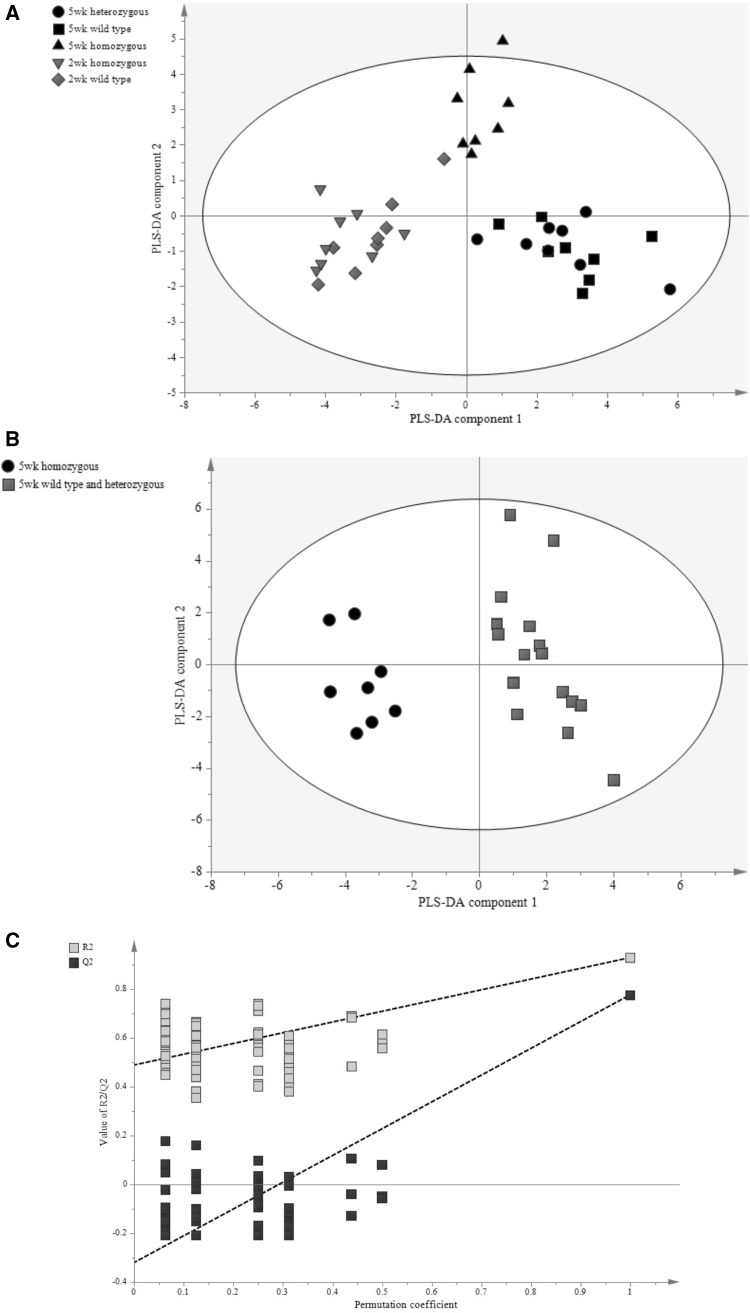

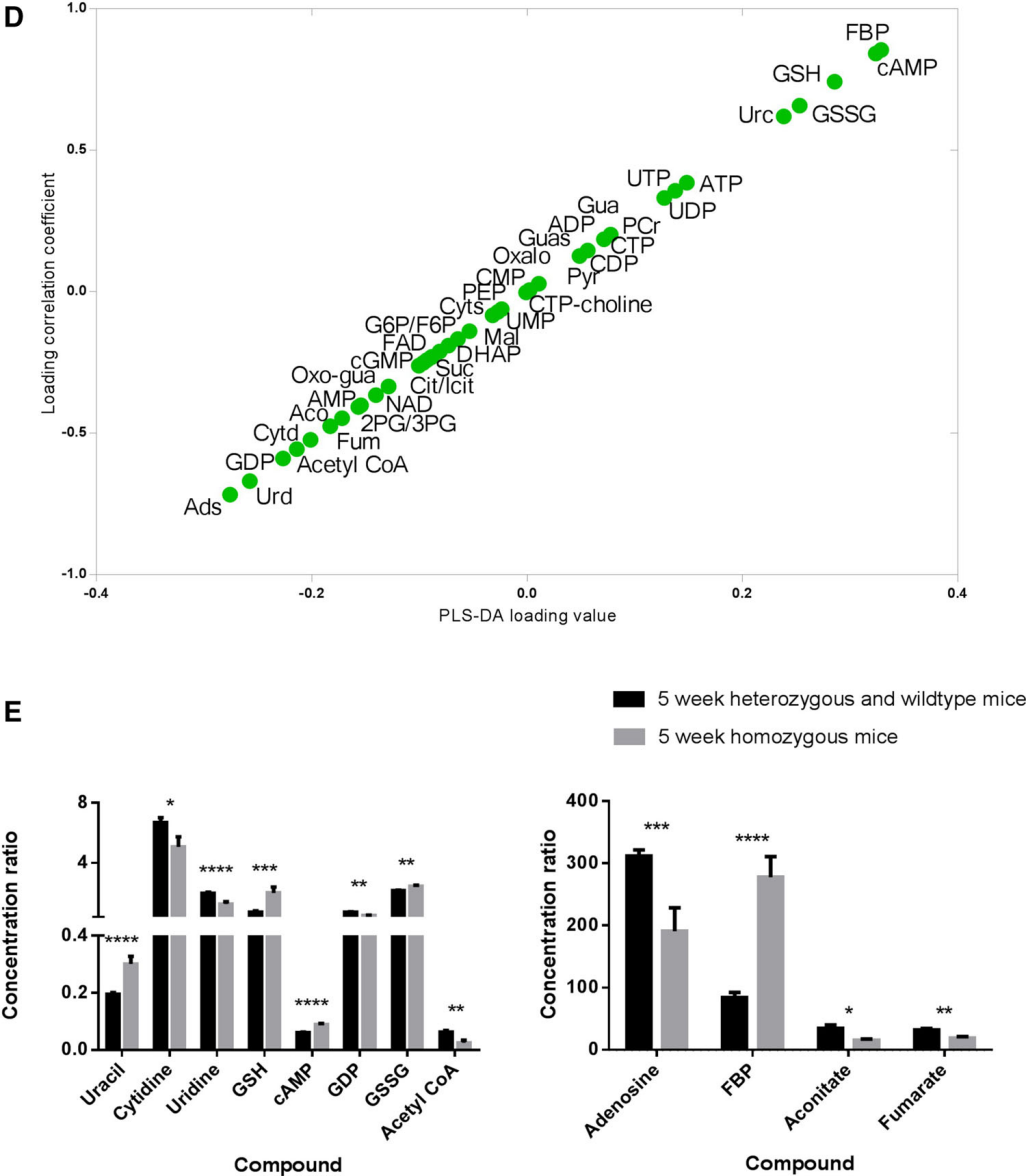
**a** Scores plot comparing profiles of chromatograms from HILIC mode aqueous analysis of tissue from wildtype, heterozygous and homozygous LMNA mouse hearts at 2 and 5 weeks (R^2^X = 31 %, R^2^Y = 41 %, Q^2^ = 20 %). **b** Scores plot comparing profiles of chromatograms from HILIC mode aqueous analysis of tissue from wildtype and heterozygous mice with homozygous laminopathic mouse hearts at the 5 week time point (R^2^X = 41 %, R^2^Y = 93 %, Q^2^ = 78 %). **c** Validation plot showing how the values of Q^2^ and R^2^ are affected by 100 random class assignments. Positive slopes of the resulting best fit *lines* indicate that random class assignment has failed to produce as significant a model as the original model. **d** An S-plot showing the contribution of the various metabolites measured to the separation between the two classes in terms of HILIC mode profiles. Metabolites in the *top right hand* corner are relatively increased in the wild type and heterozygous group and those in the *bottom left hand* corner are decreased. *FBP* fructose-1,6-bisphophate, *cAMP* cyclic AMP, *GSH* reduced glutathione, *GSSG* oxidized glutathione, *Urc* uracil, *Gua* guanine, *PCr* phosphocreatine, *Guas* guanosine, *Oxalo* oxaloacetate, *Pyr* pyruvate, *Cyts* cytosine, *PEP* phosphenol pyruvate, *G6P* glucose-6-phosphate, *F6P* fructose-6-phosphate, *Mal* malate, *Cit* citrate, *Icit* isocitrate, *Oxo-gua* oxo-guanine, *Aco* aconitate, *Cytd* cytidine, *Fum* fumarate, *Urd* uridine, *Ads* adenosine. **e** *Histograms* summarising the significant metabolic changes between the homozygous and the heterozygous and wild type mice when analysed for HILIC mode compounds. Standard *error bars* are shown and Student’s *t* tests have been carried out (*p < 0.05, **p < 0.01, ***p < 0.001, ****p < 0.0001)

The methods detailed above were applied to the analysis heart extracts from the Lmna knockout mouse, comparing animals at 2 and 5 weeks of age. A total of 39 metabolites were above the level of detection in both negative and positive ion mode using HILIC chromatography and the dataset was analysed by PLS-DA. While there was no discrimination between the 2 week old animals, the 5 week homozygous mice were readily discriminated from the 5 week wildtype and heterozygous mice (Fig. 3a). PLS-DA component 1 was associated with the difference between 2 and 5 week old animals whereas the second PLS-DA component described the genetic variation at week 5. To specifically probe genotype changes a PLS-DA model was built that compared the wild type mice and heterozygous mice with the homozygous group at the 5 week time point (Fig. 3b; Q^2^ = 78 %) and passed cross-validation (Fig. 3c). Metabolic changes are summarised in an S-plot in Fig. 3d. These changes were confirmed by univariate statistics (Fig. 3e) and associated with increases in the concentrations of uracil, glutathione (both reduced and oxidized), cAMP and fructose bisphosphate and decreases in the concentration of cytidine, uridine, GDP, acetyl-CoA, adenosine, aconitate and fumarate in the homozygous mouse. However, examining the heterozygous and wildtype samples from 40 week old mice no significant PLS-DA model could be built (data not shown). While the data used in this study were processed in a semi-quantitative manner, normalising peak areas to a labelled standard (8 deuterated carnitine species for the carnitine assay, deoxy glucose 6 phosphate for the glycolytic intermediates and [U–^13^C, ^15^N] glutamate for all other compounds with the butylated analogue of this internal standard being used for the normalisation of the amino acids) but not calculating specific concentrations, the methods we detail could have been made quantitative as they rely on the isotope dilution approach, and we were only impeded in this by the lack of availability of cheap isotopically labelled standards for metabolites.

**Fig. 4:**
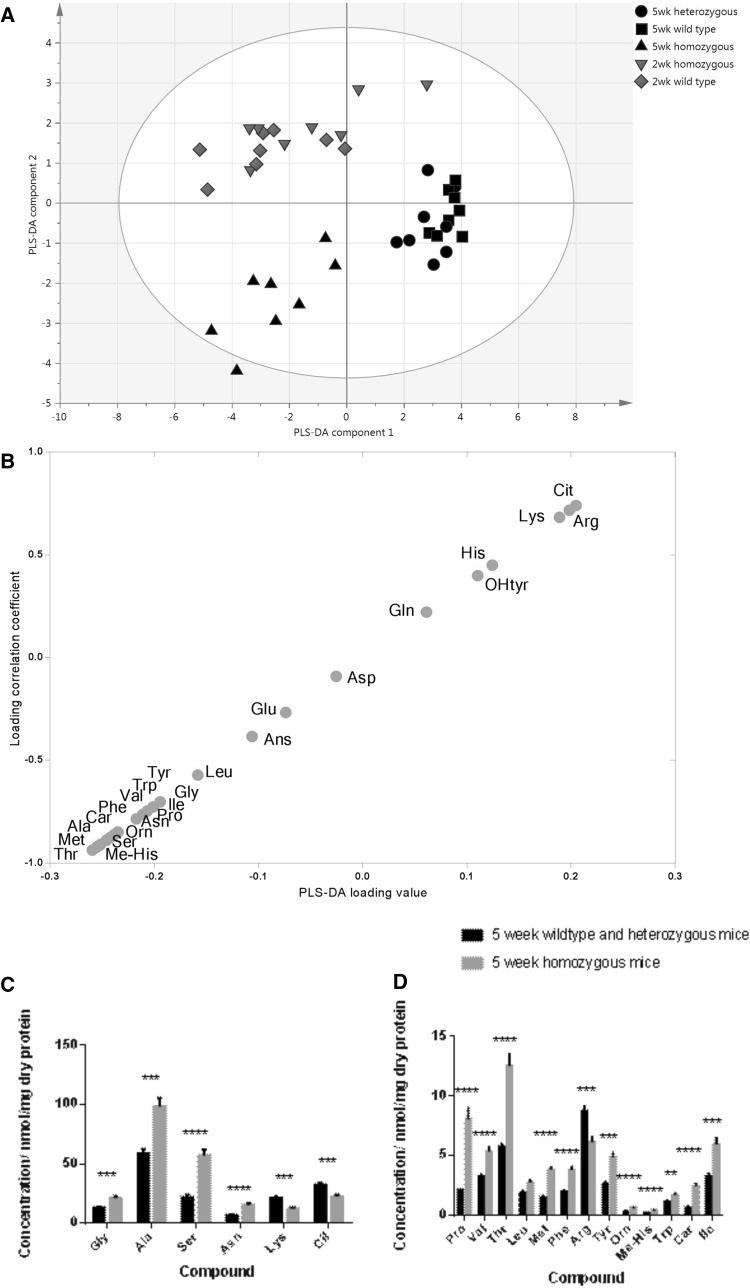
**a** PLS-DA scores plot comparing the profile of amino acids in tissue extracts from wildtype, heterozygous and homozygous laminopathic mouse hearts from mice at 2 and 5 weeks (model parameters R^2^X = 82 %, R^2^Y = 63 %, Q^2^ = 43 %). **b** An S-plot showing the contribution of the various metabolites measured in heart tissue to the separation between homozygous and the control group (wildtype and heterozygous animals). Metabolites in the top right hand corner are relatively increased in the wild type and heterozygous group and those in the bottom left hand corner are decreased. *cit* citrulline, *arg* arginine, *lys* lysine, *his* histidine, *OHtyr* hydroxy-tyrosine, *gln* glutamine, *asp* aspartate, *glu* glutamate, *ans* anserine, *leu* leucine, *tyr* tyrosine, *gly* glycine, *Ile* isoleucine, *val* valine, *trp* tryptophan, *pro* proline, *asn* asparagine, *phe* phenylalanine, *orn* ornithine, *car* carnosine, *ala* alanine, *ser* serine, *Me-His* methyl histidine, *met* methionine, *thr* threonine. **c** *Histograms* summarising the significant metabolic changes in amino acid profiles between the homozygous and heterozygous 5-week old laminopathic mouse hearts. Standard *error bars* are shown for Student’s *t* tests (*p < 0.05, **p < 0.01, ***p < 0.001, ****p < 0.0001). **d** *Histogram* showing changes in total carnitine concentrations on comparison of mouse heart tissue from wildtype, heterozygous and homozygous laminopathic mice. Standard *error bars* for Student’s *t* tests are shown. (*p < 0.05, **p < 0.01, ***p < 0.001, ****p < 0.0001)

The laminopathy model was further investigated using the amino acid analytical method described above. The same pattern was observed as for the HILIC mode in the week 2 and week 5 old mice, where one multivariate component discriminated 2 and 5 week old animals, and the other described the metabolic changes due to the genetic modification (Fig. 4a). Wildtype and heterozygous animals were treated as one group and compared to the homozygous mice using PLS-DA yielding the S-plot shown in Fig. 4b. The separation was associated with relative increases in a range of amino acids in the heart tissue of homozygous animals including alanine, serine, glycine, asparagine, proline, valine, threonine, leucine, methionine, phenylalanine and tyrosine and decreases in the concentration of arginine, citrulline, lysine and hydroxyl-tyrosine. The PLS-DA model passed cross-validation by random permutation. Extracts from the week 40 old animals could not be readily distinguished using PLS-DA and the model had a poor Q^2^**(**model parameters R^2^X = 36 %, R^2^Y = 73 %, Q^2^ = −21 %; data not shown**).** Univariate analysis was applied to the metabolites driving the changes summarised in the PLS-DA plots, demonstrating that a wide range of amino acids changed in concentration, with the majority of changes being associated with relative increases in the concentration of amino acids (Fig. 4c).

The tissue extracts were further analysed for acyl carnitines using the method described above. PLS-DA again discriminated tissue from 2 and 5 week-old animals, with one component describing the variation according to age and the other the genetic variation where the heterozygous and wildtype animals co-cluster with the homozygous animals clustering separately. Direct PLS-DA comparison of these two groups for the 5-week old animals produced a model with Q^2^ = 72 %, and analysis of the corresponding loadings plot showed that the separation was due a total decrease in carnitine concentrations in the homozygous mice relative to the combined control group (wildtype and heterozygous animals considered as a single group) and this was confirmed by univariate analysis (Fig. 4d). The same approach was applied to the week 40-old animals, but no multivariate model could be built that discriminated the two groups (data not shown).

Several groups in the field of metabolomics have attempted to develop single column methods to analyse the largest proportion of the aqueous metabolome in a single assay, including Luo et al. (2013) and Bajad et al. (2006) where bacterial extracts have had up to 20 % of their aqueous metabolome profiled. However, these approaches, because of their non-targeted nature, where the method represents a compromise across many classes of compounds, while convenient, do not produce the best quality data in terms of limits of detection and reproducibility for a large proportion of their analytical targets. Furthermore, for some species, these approaches have failed to measure the desired compounds since full chromatographic resolution is sometimes necessary in cases where ESI source fragmentation degrades analytes into metabolic precursors. We decided to sub-divide analytes into a small number of targeted assays to represent a wide proportion of cardiac metabolism, allowing for better sensitivity, reproducibility, quantification and reliability across this assay.

Several analytes of interest are too polar to be well retained and separated using a reverse phase approach and so a HILIC method was used. HILIC uses largely organic mobile phases with aqueous buffers (up to about 50 % in the eluent) as the strong solvent. An aqueous layer is formed on the surface of the silica and this layer allows retention of the analytes. The interaction between mobile phase and analyte is therefore weak enough to allow relatively unstable molecules such as ATP and other metabolites with a labile phosphate to be analysed. Most HILIC columns use a silica diol functionalisation to trap this aqueous layer on the silica particles. This was found to be unsatisfactory in the present study as it led to significant peak tailing. Zwitterionic stationary phases, as occur in ZIC-HILIC columns, were found to be more desirable as they do not exhibit tailing of more polar compounds. However, the ZIC-HILIC column used in the paper was highly susceptible to ionic contamination from the samples, causing peak tailing over continued use with sometimes as few as 100 injections of heart tissue. While Solid Phase Extraction clean-up of contaminants or column regeneration could have been employed both increase sample preparation times and neither were favourable in our pilot studies.

The application of metabolomics to studying either HCM or DCM has been relatively rare in the literature to date, despite a compelling case being made for the application of systems medicine approaches to these diseases, particularly for treatment (Piran et al. 2012). Alexander et al. (2011) used a combination of GC–MS and LC–MS to profile metabolites in the blood plasma of individuals with primary DCM, with patients with DCM being associated with decreased concentrations of steroids, glutamine, threonine and histidine, while there were increased concentrations of citric acid cycle and β-oxidation intermediates. In particular, they reported a reduction in glutamine and increased concentrations of 3-methylhistidine and prolylhydroxyproline, suggestive of increased myofibrillar and collagen degradation in DCM patients. In the present study, while glutamine did not change in the heart of the LMNA^−/−^ mouse, there were increases in both proline and methyl-histidine in the present study, along with a net increase in many amino acids, suggestive of increased myofibrillar and collagen degradation. There was also a decrease in citric acid cycle intermediates and carnitine derivatives in the heart tissue of the LMNA^−/−^ mice which suggests that in the study by Alexander et al. (2011) the increased blood plasma concentrations of citric acid cycle and β-oxidation intermediates is associated with loss from heart and muscle tissue, as a result of cellular stress and disruption of the cell membrane stability arising from the laminopathy. Similarly, Maekawa et al. (2013) observed reduced concentrations of citric acid cycle, glycolytic and pentose phosphate pathway intermediates in J2N-k cardiomyopathic hamsters, underlying the reduction in energy substrates in the DCM heart. While Maekawa et al. reported a mild decrease in glutathione in their model, and we detected a net increase in glutathione these could represent a response to ROS production in both models, with the net increase in the LMNA^−/−^ mouse suggesting that the heart has adaptively upregulated the production of the anti-oxidant to counter ROS exposure.

Examining heart tissue from patients with either chronic ischaemic (ISCM) or idiopathic dilated cardiomyopathy (IDCM) by metabolomics and proteomics, Klawitter et al. (2013) also observed a decrease in the concentration of citric acid cycle intermediates, as well as glycolysis and the malate-aspartate shuttle. While ketone bodies were increased in ISCM compared with IDCM, the metabolomics results of both our study and Klawitter et al. (2013) demonstrate profound metabolic deficits associated with DCM. Intriguingly, Klawitter et al. also highlighted changes in the phosphorylation of AKT in their proteomic dataset. As AKT is central to muscle biogenesis and protein synthesis, the changes may reflect increased protein turnover in DCM and explain the relative increase in the majority of amino acids detected in our present study. Furthermore, Mariño et al. (2014) made the interesting connection between maladaptive autophagy, cardiac remodelling and DCM that could explain the large number of changes in amino acid metabolism following cardiac remodelling associated with DCM.

The assays presented here could equally be applied in the clinical setting to monitor disease progression in those with cardiac disease. Although we primarily developed the assays to investigate how metabolism is perturbed in a mouse model of DCM, the identified metabolic changes could be used together as a fingerprint of the disease process to monitor disease progression or even treatment efficacy. A combination of metabolic changes will probably be more robust than single metabolic ‘biomarkers’ as they better represent the complex perturbations induced during human disease. It should be noted that triple quadrupoles, like the one used in the present study, are found in many hospitals, particularly for inborn error screening, and thus much of the infrastructure required to extend this approach into the clinic is already available. Finally, we also made the distinction between open and targeted metabolomic assays in the introduction, as well as the pros and cons between the two approaches. However, it should be stated that the discrimination is becoming blurred. With the advent of in silico approaches for reconstructing tandem mass spectrometry approaches such as MS^E^ (where E is the collision energy) (Zhang et al. 2009) and SWATH-MS (where a swath is cycle through consecutive precursor isolation windows) (Gillet et al. 2012) it is now possible to reconstruct such targeted analyses from untargeted approaches, and indeed detect knowns and unknowns in a simultaneous analysis.

## Concluding remarks

In conclusion, we present a quantitative method for cardiac metabolomics which covers the core metabolism of the heart, and demonstrate its applicability in terms of profiling a mouse model of DCM. The assay demonstrates that the LMNA^−/−^ mouse heart has decreased metabolites associated with the citric acid cycle and β-oxidation, but increased turnover of proteins and responses to oxidative stress.

## Electronic supplementary material

Supplementary Fig. 1 Linear responses of metabolites measured by the targeted analysis.. Supplementary material 1 (JPEG 401 kb)

Supplementary Table 1 ChEBI Identifiers for Each Metabolite Detected in the Assays. All data has been deposited to the MetaboLights database (http://www.ebi.ac.uk/metabolights/). Supplementary material 2 (DOCX 18 kb)

## References

[CR1] Alexander D, Lombardi R, Rodriguez G, Mitchell MM, Marian AJ (2011). Metabolomic distinction and insights into the pathogenesis of human primary dilated cardiomyopathy. European Journal of Clinical Investigation.

[CR2] Bajad SU, Lu W, Kimball EH, Yuan J, Peterson C, Rabinowitz JD (2006). Separation and quantitation of water soluble cellular metabolites by hydrophilic interaction chromatography-tandem mass spectrometry. Journal of Chromatography A.

[CR3] Bennett BD, Kimball EH, Gao M, Osterhout R, Van Dien SJ, Rabinowitz JD (2009). Absolute metabolite concentrations and implied enzyme active site occupancy in *Escherichia coli*. Nature Chemical Biology.

[CR4] Chance DH, Hillman SL, Millington DS, Kahler SG, Adam BW, Levy HL (1996). Rapid diagnostic of homo-cystinuria and other hypermethioninemias from newborns’ blood spots by tandem mass spectrometry. Clinical Chemistry.

[CR5] Charniot JC, Sutton A, Bonnefont-Rousselot D, Cosson C, Khani-Bittar R, Giral P (2011). Manganese superoxide dismutase dimorphism relationship with severity and prognosis in cardiogenic shock due to dilated cardiomyopathy. Free Radical Research.

[CR6] Dolinsky VW, Dyck JR (2006). Role of AMP-activated protein kinase in healthy and diseased hearts. American Journal of Physiology Heart and Circulatory Physiology.

[CR7] Dunn WB, Broadhurst D, Brown M, Baker PN, Redman CW, Kenny LC (2008). Metabolic profiling of serum using ultra performance liquid chromatography and the LTQ-Orbitrap mass spectrometry system. Journal of Chromatography B: Analytical Technologies in the Biomedical and Life Sciences.

[CR8] Feinendegen LE, Henrich MM, Kuikka JT, Thompson KH, Vester EG, Strauer B (1995). Myocardial lipid turnover in dilated cardiomyopathy: A dual in vivo tracer approach. Journal of Nuclear Cardiology.

[CR9] Fiehn O (2002). Metabolomics—The link between genotypes and phenotypes. Plant Molecular Biology.

[CR10] Gieger C, Geistlinger L, Altmaier E, Hrabé de Angelis M, Kronenberg F, Meitinger T (2008). Genetics meets metabolomics: A genome-wide association study of metabolite profiles in human serum. PLoS Genetics.

[CR11] Gillet LC, Navarro P, Tate S, Röst H, Selevsek N, Reiter L (2012). Targeted data extraction of the MS/MS spectra generated by data-independent acquisition: A new concept for consistent and accurate proteome analysis. Molecular & Cellular Proteomics.

[CR12] Goodacre R, Vaidyanathan S, Dunn WB, Harrigan GG, Kell DB (2004). Metabolomics by numbers: Acquiring and understanding global metabolite data. Trends in Biotechnology.

[CR13] Griffin JL, Atherton H, Shockcor J, Atzori L (2011). Metabolomics as a tool for cardiac research. Nature Reviews Cardiology.

[CR14] Heather LC, Wang X, West JA, Griffin JL (2013). A practical guide to metabolomic profiling as a discovery tool for human heart disease. Journal of Molecular and Cellular Cardiology.

[CR15] Hodson MP, Dear GJ, Roberts AD, Haylock CL, Ball RJ, Plumb RS (2007). A gender-specific discriminator in Sprague-Dawley rat urine: The deployment of a metabolic profiling strategy for biomarker discovery and identification. Analytical Biochemistry.

[CR16] Judge DP (2009). Use of genetics in the clinical evaluation of cardiomyopathy. JAMA.

[CR17] Kind T, Wohlgemuth G, Lee DY, Lu Y, Palazoglu M, Shahbaz S (2009). FiehnLib: Mass spectral and retention index libraries for metabolomics based on quadrupole and time-of-flight gas chromatography/mass spectrometry. Analytical Chemistry.

[CR18] Kitajima N, Watanabe K, Morimoto S, Sato Y, Kiyonaka S, Hoshijima M (2011). TRPC3-mediated Ca^2+^ influx contributes to Rac1-mediated production of reactive oxygen species in MLP-deficient mouse hearts. Biochemical and Biophysical Research Communications.

[CR19] Klawitter J, Klawitter J, Agardi E, Corby K, Leibfritz D, Lowes BD (2013). Association of DJ-1/PTEN/AKT- and ASK1/p38-mediated cell signalling with ischaemic cardiomyopathy. Cardiovascular Research.

[CR20] Le Belle JE, Harris NG, Williams SR, Bhakoo KK (2002). A comparison of cell and tissue extraction techniques using high-resolution ^1^H-NMR spectroscopy. NMR in Biomedicine.

[CR21] Lewis GD, Wei R, Liu E, Yang E, Shi X, Martinovic M (2008). Metabolite profiling of blood from individuals undergoing planned myocardial infarction reveals early markers of myocardial injury. The Journal of Clinical Investigation.

[CR22] Lu D, Ma Y, Zhang W, Bao D, Dong W, Lian H (2012). Knockdown of cytochrome P450 2E1 inhibits oxidative stress and apoptosis in the cTnT(R141W) dilated cardiomyopathy transgenic mice. Hypertension.

[CR23] Luo F, Lu R, Zhou H, Hu F, Bao G, Huang B (2013). Metabolic effect of an exogenous gene on transgenic Beauveria bassiana using liquid chromatography-mass spectrometry-based metabolomics. Journal of Agriculture and Food Chemistry.

[CR24] Maekawa K, Hirayama A, Iwata Y, Tajima Y, Nishimaki-Mogami T, Sugawara S (2013). Global metabolomic analysis of heart tissue in a hamster model for dilated cardiomyopathy. Journal of Molecular and Cellular Cardiology.

[CR25] Mariño G, Pietrocola F, Kong Y, Eisenberg T, Hill JA, Madeo F (2014). Dimethyl α-ketoglutarate inhibits maladaptive autophagy in pressure overload-induced cardiomyopathy. Autophagy.

[CR26] Mayr M (2008). Metabolomics: Ready for the prime time?. Circulation: Cardiovascular Genetics.

[CR27] Nakamura T, Sugihara H, Kinoshita N, Yoneyama S, Azuma A, Nakagawa M (2000). Can serum carnitine levels distinguish hypertrophic cardiomyopathy from hypertensive hearts?. Hypertension.

[CR28] Nicholson JK, Lindon JC, Holmes E (1999). ‘Metabonomics’: Understanding the metabolic responses of living systems to pathophysiological stimuli via multivariate statistical analysis of biological NMR spectroscopic data. Xenobiotica.

[CR29] Piran S, Liu P, Morales A, Hershberger RE (2012). Where genome meets phenome: Rationale for integrating genetic and protein biomarkers in the diagnosis and management of dilated cardiomyopathy and heart failure. Journal of the American College of Cardiology.

[CR30] Razeghi P, Young ME, Ying J, Depre C, Uray IP, Kolesar J (2002). Downregulation of metabolic gene expression in failing human heart before and after mechanical unloading. Cardiology.

[CR31] Stadtman ER, Levine RL (2003). Free radical-mediated oxidation of free amino acids and amino acid residues in proteins. Amino Acids.

[CR32] Sullivan T, Escalante-Alcalde D, Bhatt H, Anver M, Bhat N, Nagashima K (1999). Loss of A-type lamin expression compromises nuclear envelope integrity leading to muscular dystrophy. Journal of Cell Biology.

[CR33] Taha M, Lopaschuk GD (2007). Alterations in energy metabolism in cardiomyopathies. Annals of Medicine.

[CR34] Tautenhahn R, Cho K, Uritboonthai W, Zhu Z, Patti GJ, Siuzdak G (2012). An accelerated workflow for untargeted metabolomics using the METLIN database. Nature Biotechnology.

[CR35] Taylor MR, Carniel E, Mestroni L (2006). Cardiomyopathy, familial dilated. Orphanet Journal of Rare Diseases.

[CR36] Wang TJ, Larson MG, Vasan RS, Cheng S, Rhee EP, McCabe E (2011). Metabolite profiles and the risk of developing diabetes. Nature Medicine.

[CR37] Wang-Sattler R, Yu Z, Herder C, Messias AC, Floegel A, He Y (2012). Novel biomarkers for pre-diabetes identified by metabolomics. Molecular Systems Biology.

[CR38] Wishart DS (2009). Computational strategies for metabolite identification in metabolomics. Bioanalysis.

[CR39] Zhang T, Creek DJ, Barrett MP, Blackburn G, Watson DG (2012). Evaluation of coupling reversed phase, aqueous normal phase, and hydrophilic interaction liquid chromatography with Orbitrap mass spectrometry for metabolomic studies of human urine. Analytical Chemistry.

[CR40] Zhang H, Grubb M, Wu W, Josephs J, Humphreys WG (2009). Algorithm for thorough background subtraction of high-resolution LC/MS data: Application to obtain clean product ion spectra from nonselective collision-induced dissociation experiments. Analytical Chemistry.

