# Supplementary figures and images for: A targeted metabolomics assay for cardiac metabolism and demonstration using a mouse model of dilated cardiomyopathy
Source: Metabolomics. 2016 Mar 7;12:59. doi: 10.1007/s11306-016-0956-2 (PMC4781888; doi:10.1007/s11306-016-0956-2)

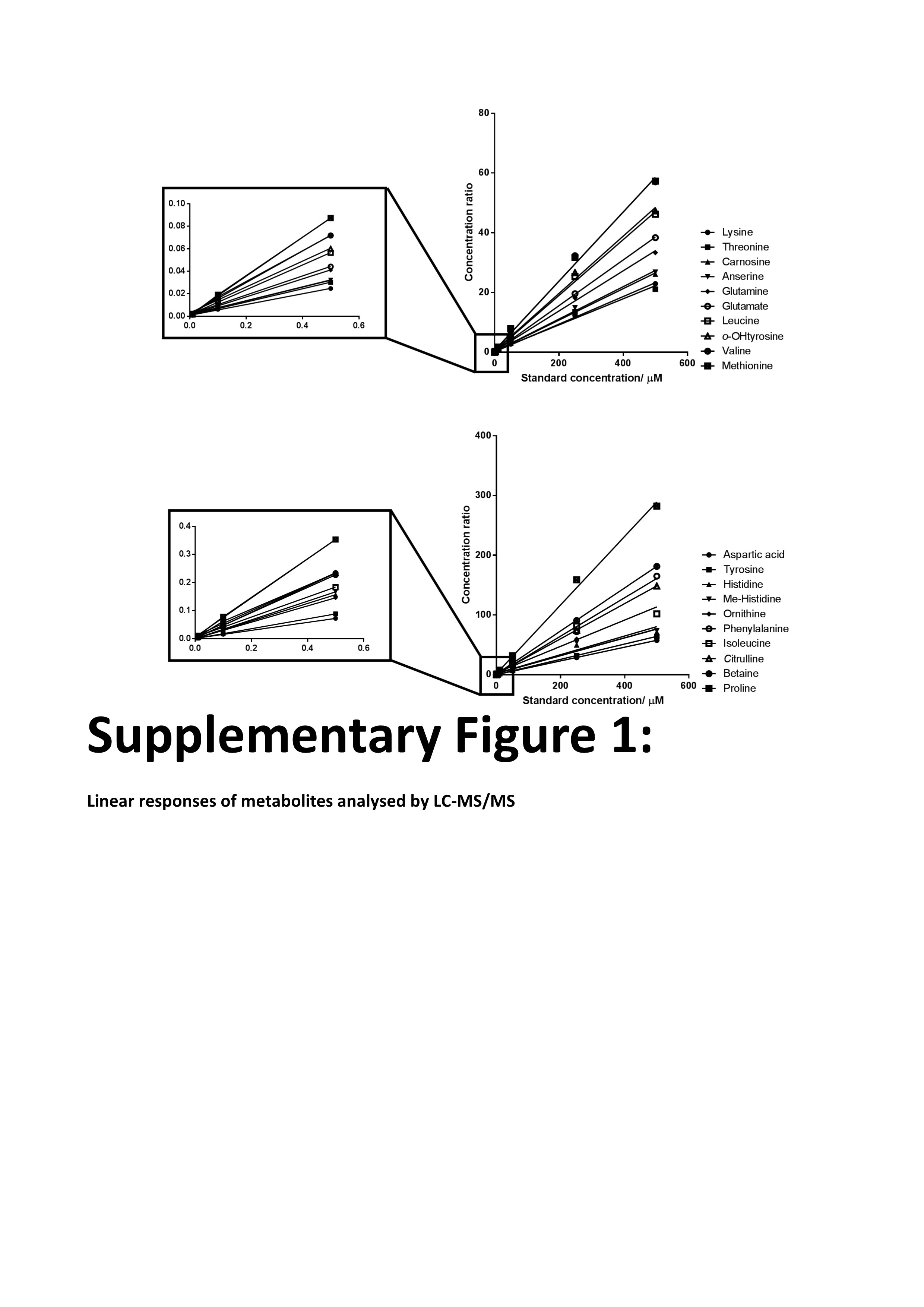

Supplement: Supplementary file 1 — Linear responses of metabolites measured by the targeted analysis.. Supplementary material 1 (JPEG 401 kb) [file 11306_2016_956_MOESM1_ESM.jpg]
