# Supplementary material for: A targeted metabolomics assay for cardiac metabolism and demonstration using a mouse model of dilated cardiomyopathy
Source: Metabolomics. 2016 Mar 7;12:59. doi: 10.1007/s11306-016-0956-2 (PMC4781888; doi:10.1007/s11306-016-0956-2)
Supplement: Supplementary file 2 — ChEBI Identifiers for Each Metabolite Detected in the Assays. All data has been deposited to the MetaboLights database (http://www.ebi.ac.uk/metabolights/). Supplementary material 2 (DOCX 18 kb) [file 11306_2016_956_MOESM2_ESM.docx]

**Supplementary Table 1: ChEBI Identifiers for the Metabolites Assayed**

<http://www.ebi.ac.uk/chebi/init.do>

All metabolites have been confirmed by use of authentic standards during the development of the assay, except for the carnitines where carnitines not available in the commercial standard were confirmed by mass and chromatography measurements. Thus, assignments are to level 1 of the Metabolomics Standards Initiative.

**Name ChEBI Identifier**

| 2-phosphoglycerate 24344 |
| --- |
| 3-phosphoglycerate 61304 |
| Acetyl CoA 15351 |
| Aconitate 22210 |
| Adenine 16708 |
| Adenosine 16335 |
| S-Adenosyl L-methionine 15414 |
| ADP 16761 |
| Alanine 16977 |
| AMP 456215 |
| Anserine 18323 |
| L-Arginine 16467 |
| L-Asparagine 17196 |
| Aspartate 29993 |
| ATP 15422 |
| Glycine betaine 17750 |
| C10 carnitine **28717** |
| C10:1 carnitine 73048 |
| C10:2 carnitine **17387*** |
| C12 carnitine **17387*** |
| C12:1 carnitine **85446** |
| C14 carnitine 84634 |
| C14:1 carnitine **85449** |
| C14:2 carnitine **17387*** |
| C14-OH carnitine **17387*** |
| C16 carnitine 73067 |
| C16:1 carnitine **85455** |
| C16:1-OH carnitine **85456** |
| C16:2 carnitine **17387*** |
| C16-OH carnitine **85519** |
| C18 carnitine **84644** |
| C18:1 carnitine **84651** |
| C18:1-OH carnitine **17387*** |
| C18:2 carnitine **84098** |
| C18:2-OH carnitine 73075 |
| C18-OH carnitine 73076 |
| C2 carnitine **73024** |
| C20 carnitine butyl ester 73100 |
| C20:1 carnitine 73118 |
| C20:2 carnitine 73119 |
| C3 carnitine **28867** |
| C4 carnitine **17387*** |
| C4 dicarboxyl carnitine 73034 |
| C5 carnitine butyl ester 85095 |
| C5 dicarboxyl carnitine **17387*** |
| C5:1 carnitine **17387*** |
| C5-OH carnitine **17387*** |
| C6 carnitine **85519** |
| C6 dicarboxyl carnitine **17387*** |
| C8 carnitine 73039 |
| C8 dicarboxyl carnitine 73052 |
| C8:1 carnitine 73037 |
| C8-OH carnitine 84100 |
| cAMP 17489 |
| Carnosine 15727 |
| CDP 17239 |
| CDP-choline 58779 |
| cGMP 16356 |
| Citrate 35808 |
| L-Citrulline 16349 |
| CMP 17361 |
| CTP 37563 |
| L-Cystine 17561 |
| Cytidine 17562 |
| Cytosine 16040 |
| Dihydroxyacetonephosphate 57642 |
| FAD 16238 |
| Free carnitine 17126 |
| D-Fructose 1,6-bisphosphate 78682 |
| Fumarate 29806 |
| GDP 58189 |
| L-glutamine 18050 |
| L-glutamate 29988 |
| D-Glucose 6 phosphate/ D-Fructose 6 phosphate 58225 |
| Glycine 15428 |
| GMP 17345 |
| GSH 16856 |
| GSSG 58297 |
| GTP 57600 |
| Guanine 16235 |
| Guanosine 16750 |
| L-Hisitdine 15971 |
| L-Leucine & L-Isoleucine 15603 & 17191 |
| L-Lysine 18019 |
| L-Malate 15589 |
| Malonyl CoA 15531 |
| L-Methionine 16643 |
| Methyl Cytosine 27551 |
| Methyl Histidine 70958 & 70959 |
| NAD 15846 |
| NADP 16474 |
| *o*-Hydroxy Tyrosine |
| *o-*Nitro tyrosine |
| L-Ornothine 15729 |
| Oxaloacetate 16452 |
| L-methionine S-oxide 17016 |
| Phosphocreatine 17287 |
| Phosphoenolpyruvate 18021 |
| L-Phenylalanine 17295 |
| L-Proline 17203 |
| Pyruvate 15361 |
| *S*-adenosyl-*L*-homocysteine 16680 |
| L-Serine 74819 |
| L-Threonine 16857 |
| L-Tryptophan 16828 |
| L-Tyrosine 17895 |
| UDP 17659 |
| UMP 57865 |
| Uracil 17568 |
| Uridine 16704 |
| UTP 57481 |
| Valine 16414 |
| α ketoglutarate 16810 |

***17387 signifies a generic acyl-carntine where a specific species cannot be found in the ChEBI database.**
